# Supplementary material for: Iterative improvement in the automatic modular design of robot swarms
Source: PeerJ Comput Sci. 2020 Dec 7;6:e322. doi: 10.7717/peerj-cs.322 (PMC7924708; doi:10.7717/peerj-cs.322)
Supplement: Supplemental Information 3 [file peerj-cs-06-322-s003.zip › argos3/doc/api/standalone/a00593_source.html]

ARGoS: plugins/simulator/entities/wheeled\_entity.h Source File


- Main Page
- Related Pages
- Namespaces
- Classes
- Files

- File List
- File Members

# plugins/simulator/entities/wheeled\_entity.h

Go to the documentation of this file.

```
00001 
00007 #ifndef WHEELED_ENTITY_H
00008 #define WHEELED_ENTITY_H
00009 
00010 #include <argos3/core/simulator/entity/entity.h>
00011 #include <argos3/core/utility/math/vector3.h>
00012 
00013 namespace argos {
00014 
00015    class CWheeledEntity : public CEntity {
00016 
00017    public:
00018 
00019       ENABLE_VTABLE();
00020 
00021    public:
00022 
00023       CWheeledEntity(CComposableEntity* pc_parent,
00024                      size_t un_num_wheels);
00025 
00026       CWheeledEntity(CComposableEntity* pc_parent,
00027                      const std::string& str_id,
00028                      size_t un_num_wheels);
00029 
00030       virtual ~CWheeledEntity();
00031 
00032       virtual void Reset();
00033 
00034       inline size_t GetNumWheels() const {
00035          return m_unNumWheels;
00036       }
00037 
00038       void SetWheel(UInt32 un_index,
00039                     const CVector3& c_position,
00040                     Real f_radius);
00041 
00042       const CVector3& GetWheelPosition(size_t un_index) const;
00043 
00044       inline const CVector3* GetWheelPositions() const {
00045          return m_pcWheelPositions;
00046       }
00047 
00048       Real GetWheelRadius(size_t un_index) const;
00049 
00050       inline const Real* GetWheelRadia() const {
00051          return m_pfWheelRadia;
00052       }
00053 
00054       Real GetWheelVelocity(size_t un_index) const;
00055 
00056       inline const Real* GetWheelVelocities() const {
00057          return m_pfWheelVelocities;
00058       }
00059 
00060       void SetVelocities(Real* pf_velocities);
00061 
00062       virtual std::string GetTypeDescription() const {
00063          return "wheels";
00064       }
00065 
00066    private:
00067 
00068       size_t m_unNumWheels;
00069       CVector3* m_pcWheelPositions;
00070       Real* m_pfWheelRadia;
00071       Real* m_pfWheelVelocities;
00072 
00073    };
00074 
00075 }
00076 
00077 #endif
```

---

Generated on 10 Jul 2018 for ARGoS by 
 1.6.1
